# Experiments and Observations upon the State of the Air in the Fever Hospitals of Cork, at a Time When They Were Crowded with Patients, Labouring under Febrile Contagion

**Published:** 1818-04

**Authors:** Edmund Davy

**Affiliations:** Professor of Chemistry, and Secretary to the Cork Institution.


*For the London Medical and Physical Journal.*

*Experiments and Observations upon the State of the Air in the Fever Hospitals of Cork, at a Time when they were crowded with Patients, labouring under Febrile Contagion.* By EDMUND DAVY, Esq. Professor of Chemistry, and Secretary to the Cork Institution.

[The following experiments originally appeared in the Cork Intelligencer, December 9, 1817; their accuracy and importance appeared, however, to render them proper for our Journal.]

FROM numerous experiments made on air, collected in different countries, by the most enlightened enquirers, it seems to be generally admitted, that the chemical constitution of the atmosphere is nearly the same at all seasons of the year, and in all parts of the globe. Nitrogen and oxygen gases form its principal component parts: it also contains a minute portion of carbonic acid gas, and a variable quantity of aqueous vapour. As oxygen gas is essential to animal and vegetable life, and to the processes of combustion, fermentation, &c.; and, as it is constantly entering into new forms, by which its peculiar properties are modified or destroyed, it is considered the most important, and the most active part of the atmosphere. The most general and important change that the oxygenous portion of the air undergoes is its conversion into carbonic acid gas, a substance, which, though obnoxious to animals, is yet made subservient to vegetable life; and this change is invariably connected with the exertion of the vital functions of organic beings, and with the burning of coals, wood, candles, &c. The salubrity and healthy state of the air depend, in a great measure upon the quantity of oxygen gas it contains; and, this quantity (about 21 per cent.) appears to exist in all places exposed to the free atmosphere and the influence of winds. But the same uniformity of composition does not prevail in the air of confined dwelling-houses, crowded theatres, and hospitals, that are badly ventilated. At a time when typhus was very prevalent in Cork, and there were, in the two fever hospitals, about 280 patients, labouring, for the most part, under febrile infection, it occurred to my friend Dr. Daly, whose active exertions in the cause of humanity are well known, and likewise to myself, that it would be a desirable object to ascertain the state of the air in the fever-wards; and I immediately undertook a series of experiments on the subject. To give in detail all the minutiæ of my experiments, would far exceed the limits of this paper; I shall, therefore, briefly notice my methods and results, and close the communication with a few observations connected with the subject. I procured air from five large and small wards in the House of Recovery, and from the two wards in Peacock-lane Hospital: I collected it from different parts of the rooms; as, in the middle, at the sides, near the floor, and at different heights from it, and close to the beds of the patients. In every instance, the air was obtained by emptying on the spot bottles that had been previously filled with distilled water, and immediately closing them. The bottles were perfectly air-tight, being all furnished with well-ground glass stoppers. The air was examined soon after it had been collected. The first and most important object of my inquiry, was to ascertain the quantity of oxygen gas in the several bottles of air. For this purpose, I employed hydrogen gas, and the electric spark; a method that seems to unite more simplicity and elegance than any other; and, with due precaution, is susceptible of great accuracy. As the purity of the hydrogen, used in experiments of this kind, is of consequence to the accuracy of the results, it may be proper to notice the mode by which it was obtained; especially as it has, I think, some little novelty, and seems to be quite unexceptionable, precluding all source of error, from the presence of common air. I put some small pieces of zinc into a glass, and nearly filled it with water that had been boiling for some time. I then filled a tube with the boiling water, and inverted it in the glass, and after adding sulphuric acid, I shortly after collected the gas.

I made a great number of experiments, using, in every instance, an excess of hydrogen gas. In every trial, I mixed 0·30 of a cubic inch of the air under examination with 0·30 of pure hydrogen gas; and, after agitating the mixture in a long, thick, detonating tube, furnished with wires, the charge of a Leyden phial was passed through the tube; and the residual air, on being transferred to the cubic inch measure, occupied about 0·40 of it. I venture to state this as a general result; for, though, in a few cases, there was a difference of about one per cent. more or less, yet this difference was rather apparent than real, owing to the difficulty of measuring uniform quantities of air, and it was corrected by a careful repetition of the experiments. Now, as two volumes of hydrogen and one of oxygen gas enter into the composition of water,—if the foregoing results are made the basis of a calculation,—the apparent quantity of the oxygen gas in the air from the different fever-wards will amount to about 22·22 per cent.; but this is not the real quantity; a slight allowance must be made for a minute portion of air disengaged from the water after the detonation of the mixed gases; and, when this is taken into account, the oxygen may be fairly estimated at about 21 per cent. And, according to the statements of Sir Humphry Davy, and other able chemists, 21 per cent. is the actual quantity of oxygen gas in the external atmosphere, in different parts of the globe. It may be remarked, that the variations in the temperature and pressure of the atmosphere, during the preceding experiments, were so small, as not to influence the accuracy of the general results stated.

With a view to confirm the preceding statements, I made comparative trials upon air collected from the open atmosphere, at the top of the observatory belonging to the Cork Institution; a situation, perhaps, not less salubrious than any other in Cork. The experiments were conducted in a manner precisely similar to those I have noticed; part of the same hydrogen was employed, and every precaution used to ensure accuracy. And, in every case in which the electric spark was passed through a mixture of the air under examination and hydrogen gas, in the proportion of 0·30 of each, the residual air measured about 0·40. I collected air from Hughes's-lane, a place notorious for the number of cases it had furnished of typhus; but it yielded, on examination, the same uniformity of result.

I have made some trials on the other gaseous constituents of the air, collected from the different fever-wards, and compared them with similar experiments on air from the observatory of the institution ; and, I have found a very near coincidence in both series of results.

Thus, judging from the absorption that took place in the bottles of air from the fever-wards, when placed for some time in water, and when agitated in this fluid,—and, especially from the effects of lime-water on the air,—and, comparing, by similar trials, air collected from the atmosphere in salubrious situations; I could scarcely, in either case, discover a perceptible difference in the quantity of carbonic acid gas. In one instance, I filled a two-quart ground-stoppered bottle with the air from a large ward at the House of Recovery ; and, on the spot, I put into the bottle a small phial of lime-water, and well closed it. After much occasional agitation, and an interval of about two days, I examined the carbonate of lime formed, and compared it with the quantity produced under similar circumstances from the same bottle filled with air from the observatory, and treated with lime-water ; and I was unable, in this way, to detect any appreciable difference. If this method may be relied on, I think I may venture to state, that the air from the ward did not contain near one per cent. more of carbonic acid gas than the air from the observatory.

After I had separated oxygen and carbonic acid gas from the different airs examined, I could not detect the presence of any other gas than nitrogen, which exhibited its characteristic negative properties. The want of leisure prevented me from varying and multiplying my experiments, so as to ascertain the exact proportion of the carbonic acid and nitrogen gases in the airs ; and it may be proper to observe that, during the time I was engaged in this enquiry, the variations of temperature, moisture, and pressure of the atmosphere, were very small, and too often connected with accidental circumstances, to be accurately noticed.

